# Dielectric meta-atom with tunable resonant frequency temperature coefficient

**DOI:** 10.1038/s41598-017-02974-9

**Published:** 2017-05-31

**Authors:** Lingling Wu, Xiaoqing Xi, Bo Li, Ji Zhou

**Affiliations:** 1 State Key Laboratory of New Ceramics and Fine Processing, School of Materials Science and Engineering, Tsinghua University, Beijing, 100084 China; 2 Advanced Materials Institute, Shenzhen Graduate School, Tsinghua University, Shenzhen, China

## Abstract

In this paper, we present a proof-of-concept of a new approach to achieving tailored resonant frequency temperature coefficients in dielectric meta-atoms. The technique involves introducing a thermally expanding or contracting material joining the active high permittivity dielectric absorbers. Both simulation and experiment show that by careful design of the element size and appropriate choice of thermomechanical intermediate layer material, increased or decreased resonant frequency shift temperature sensitivity is possible. Once the active dielectric material is chosen, and a meta-atom design determined, we show the resonant frequency shift depends on the thermal expansion coefficient of the intermediate layer. This work demonstrates the feasibility of manipulating the blue or red shift of metamaterial devices by introducing temperature responsive intermediate layers into meta-atoms.

## Introduction

In the past decades, electromagnetic (EM) metamaterials^1–3^ have attracted considerable enthusiasm of researchers because of their remarkable properties that could be applied to achieve abnormal permittivity or permeability^4–6^, EM cloaking^7–9^, perfect lensing^10, 11^, tunable band-pass filters^12, 13^, microwave couplers^14^, absorbers^15^ and other uses. Compared with resonant metallic elements, nonmetallic dielectric resonators show a lot of advantages in constructing metamaterials with isotropic electromagnetic responses and smaller conductive loss at high operating frequencies^16^. There are many forms of electric or magnetic resonance in dielectric materials^17, 18^, such as ferroelectrics^19^, negative permeability Mie resonance particles^20^, ferromagnetic resonance in ferrites^21^ and others. Among these, Mie resonance is a simple approach based on displacement currents^22–24^. The first order Mie resonance frequency *f*_*1*_ is given by

1 $${f}_{1}=\frac{{\theta }_{1}c}{2\pi r\sqrt{{\varepsilon }_{2}{\mu }_{2}}}$$

where *θ*_1_ is a constant close to *π*, *c* is the light velocity in vacuum, *r* is the radius of dielectric meta-atoms, *ε*_2_ and *μ*_2_ is the relative permittivity and permeability of the dielectric meta-atoms. For non-magnetic dielectric material, *μ*_2_ is usually approximately equal to 1. As suggested by equation (1), once the size of the dielectric element is fixed, the frequency of the first order Mie resonance is decided by the relative permittivity of the dielectric material. The permittivity of many important dielectric materials such as CaTiO_3_ are very sensitive to temperature^25, 26^. The frequency shift of the dielectric materials caused by temperature change is characterized by the temperature coefficient of resonant frequency (TCF), which is calculated by

2 $${\tau }_{f}=-(\frac{1}{2}{\tau }_{\varepsilon }+{\alpha }_{L})$$

where *τ*_*f*_ is the TCF, *τ*_*ε*_ is the temperature coefficient of permittivity, and *α*_*L*_ is the linear thermal expansion coefficient. Many investigations have manipulated *τ*_*f*_ to achieve desired blue or red shifts to fulfil the specific practical requirements. Several of these obtained tunable *τ*_*f*_ by mixing two of more opposite *τ*_*f*_ materials^27, 28^, such as MgTiO_3_-CaTiO_3_^29^ and Ba(Zn,Nb)O_3_-Ba(Zn,Ta)O_3_^30^. Unfortunately, the fabrication process of these composites is usually very complicated and involves high sintering temperature. Moreover, the component materials to be mixed together should be carefully selected to meet other requirement, such as high Q and proper value of *ε*, limiting their usability in meta-devices.

Therefore, a more universal approach to control TCF, applicable to most dielectric materials, is highly desirable. From equation (1), the TCF is closely connected with the coefficient of thermal expansion (CTE) of the material. This indicated to us that the TCF of materials could be manipulated by introducing a thermally responsive second component of suitable CTE into the design of the meta-atoms.

In recent years, tunable CTE artificial materials have been widely investigated, demonstrating negative-, positive- or near-zero CTEs^31–38^, offering a simple and versatile route to manipulating the EM properties of metamaterials through thermal expansion or contraction. Therefore, by constructing the meta-atoms using both the high permittivity dielectric and a second material with a desired CTE, the TCF of the EM meta-devices could be manipulated. The second material would not have an absorption resonance, but would simply act to change the spacing between the active dielectric elements.

In this paper, we use low-cost and easily accessible thermally sensitive materials in a simple design. We introduce tailored CTE joints connecting separate blocks of high permittivity dielectric. The meta-atom then consists of a tiling of dielectric and thermomechanical materials. Through simulation and experimental result, we show that the effective TCF of the meta-atom is closely related to the CTE value of the expanding or contracting joints. We will call these joints the intermediate material or equivalently the intermediate layer. We believe that this approach could be broadly applied in EM device design using tailorable TCF to meet specific performance requirements. When temperature of such a device changes, the intermediate material will shrink or expand to either cancel or enhance the effects of the permittivity variation with temperature. Permittivity changes alone cause blue shifts in the resonance frequency with increasing temperature. Thermal expansion pushing the active elements apart also blue shifts the frequency. Contraction upon heating, a negative CTE, red shifts the frequency and can in principle cancel the permittivity-induced shift. The resonant frequency could then potentially be held stable. Alternately, with a positive CTE intermediate layer, it can be given a significant chirp upon heating.

## Results

**Figure 1:**
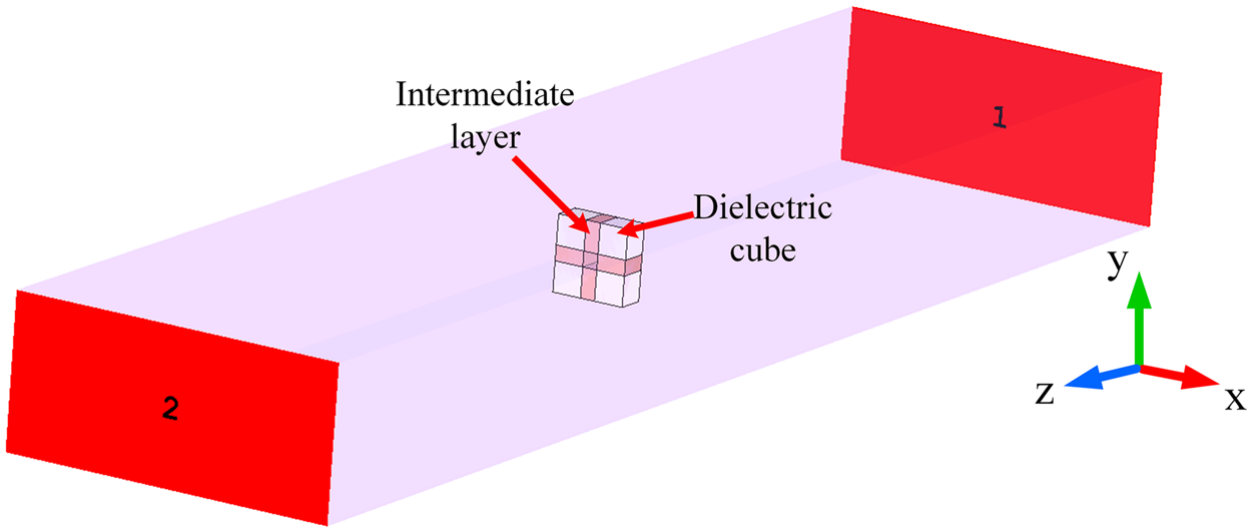
Schematic of the model meta-atom and test environment used in the simulation.

To demonstrate the feasibility of our proposed method, a meta-atom composed of four dielectric cubes and thermally variable intermediate layer is designed in this paper. To explore the relationship between the thermal expansion of the intermediate layer and the effective TCF of the meta-atom, we carried out simulation work using the Microwave Studio software package (CST Studio Suite 2016, Germany), on the model shown in Fig. 1. The dimensions of the rectangular vacuum box surrounded by a perfect conductor is 22.86 mm × 10.16 mm × 100 mm (EIA WR-90 waveguide), in which a single-mode with electric field E parallel to the y-axis from 8.2 GHz to 12.4 GHz (X-band) can be transmitted.

**Figure 2:**
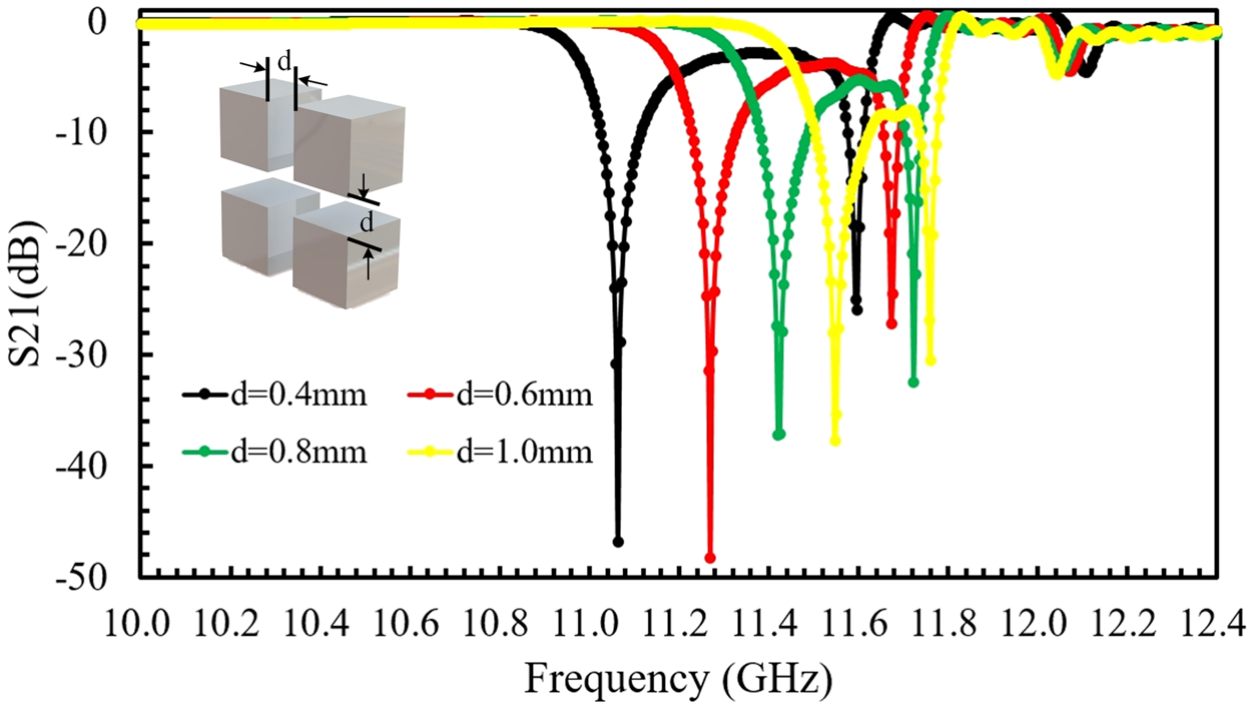
The relationship between the first order resonance frequency and the width of the intermediate layer.

We firstly simulated the relationship between the width of intermediate layer and the first order resonant frequency of the meta-atom. The meta-atom was designed with four dielectric cubes with a cross-shaped intermediate layer with a constant permittivity of 2.3. To simplify the modeling and to match experiment, the dielectric cubes have a permittivity of 120.6 at room temperature (CaTiO_3_ ceramic). The simulated result is shown in Fig. 2. The first order resonant frequency has a blue shift as the width of the intermediate layer increases from 0.4 to 1 mm.

**Figure 3:**
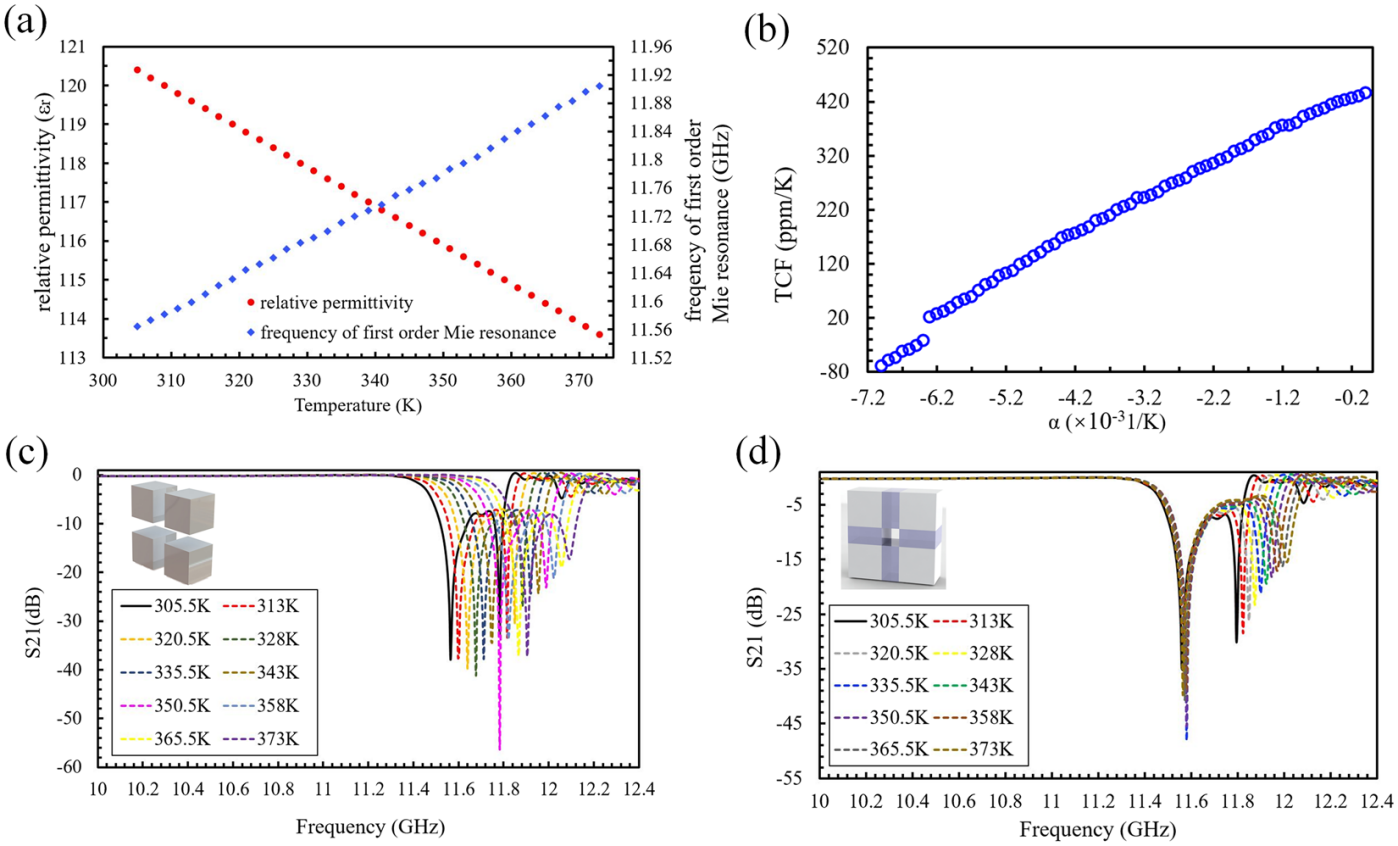
(**a**) Calculated relationship between the relative permittivity and temperature (red point curve) and the frequency of the first order Mie resonance and temperature (blue elliptical dot curve). (**b**) Calculated effective TCF of the meta-atom versus the thermal expansion coefficient of the intermediate layer. (**c,d**) The shift of the first order resonance frequency for the meta-atom (**c**) without elastic intermediate layer, and (**d**) with an intermediate layer (*α*_*in*_ = −6.35 × 10^−3^ K^−1^).

Since the CTE of dielectric cubes is very small, we neglect the thermal expansion, and only consider the expansion of intermediate layer. The permittivity of CaTiO_3_ ceramic decreases with increasing temperature in accordance with the Curie-Weisslaw, so the first resonant frequency will also have a blue shift when temperature increases, as shown in Fig. 3(a). During the simulation, the thermal expansion coefficient of the intermediate layer *α*_*in*_ is varied from −7 × 10^–3^ K^−1^ to −1 × 10^−4^ K^−1^ in steps of 1 × 10^−4^ K^−1^, and the temperature increases from 305.5 K to 373.0 K in steps of 7.5 K. The simulated frequency range is from 10.0 GHz to 12.4 GHz to coincide with the experiment. From the simulation result, the effective TCF of the meta-atom is calculated and shown in Fig. 3(b). The effective TCF of the meta-atom has an approximately linear dependence on the thermal expansion of the intermediate layer, and it is close to zero at *α*_*in*_ = −6.35 × 10^−3^ K^−1^. The scattering matrix transmission coefficient S_21_ is shown in Fig. 3(c) and (d) versus frequency at a range of temperatures. Figure 3(c) is the calculated S_21_ spectrum for the square array of four dielectric cubes alone, while in Fig. 3(d) the cubes are joined by an intermediate layer with *α*_*in*_ = −6.35 × 10^−3^ K^−1^.

**Figure 4:**
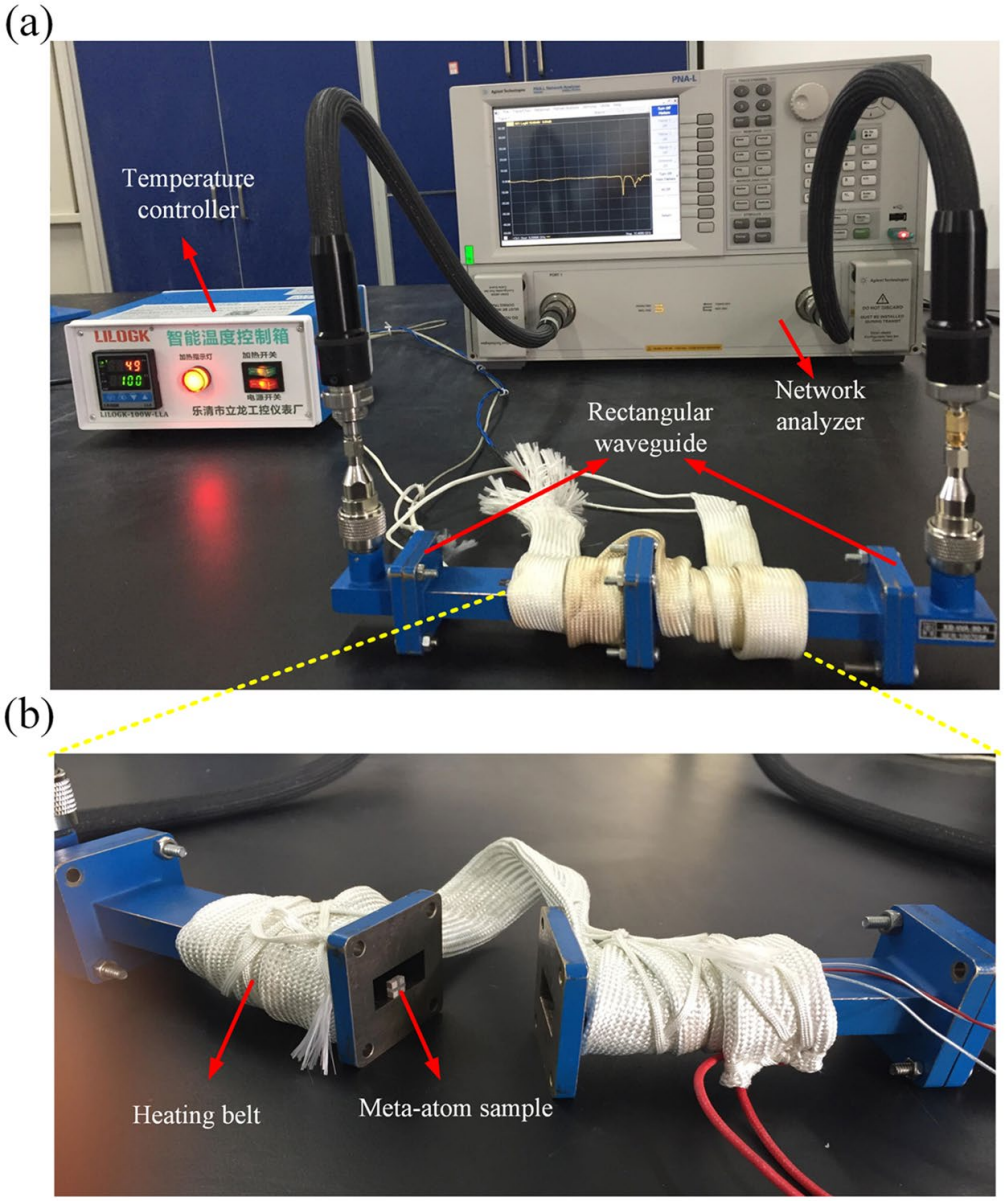
(**a**) The measurement setup. (**b**) The sample placed in the waveguide.

**Figure 5:**
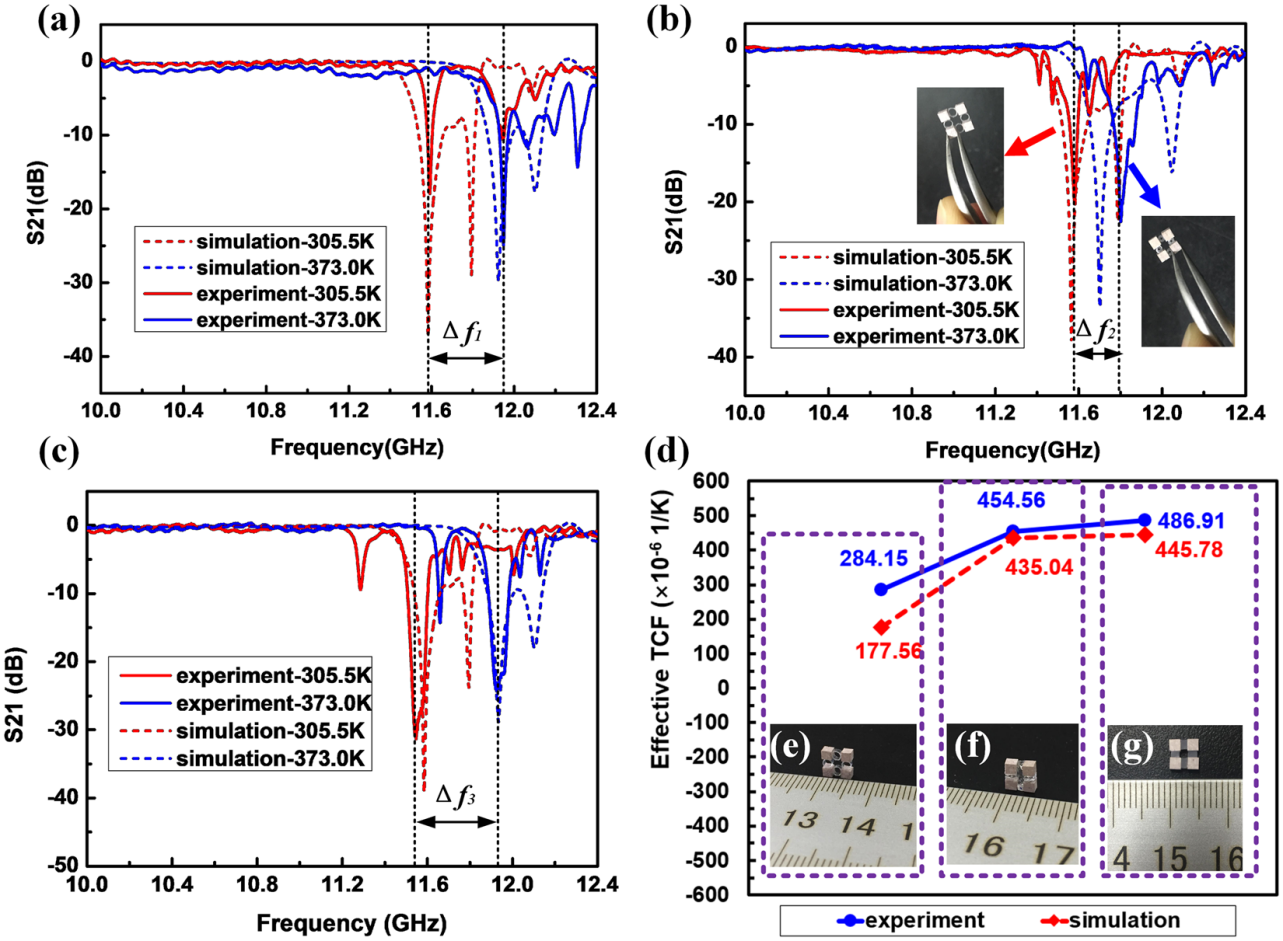
(**a**–**c**) The simulated and measurement resonance frequency shift of met-atom with intermediate layer of (**a**) silica. (**b**) heat-shrink tubing and (**c**) rubber. (**d**) The comparison of simulated and experiment effective TCF of the three meta-atoms. (**e**–**g**) Photos of the fabricated meta-atom samples. From left to right these are the heat shrink tubing layer, the silica layer, and the rubber layer. Photo insets in (**b**) show the initial shape (red arrow) of the meta-atom sample with heat-shrink tubing layer and its deformed shape (blue arrow) after heating.

The experiment measures the frequency spectrum of the transmission parameter S_21_ of three meta-atom samples. The setup is illustrated in Fig. 4(a). The dielectric samples are 2 × 2 × 2 mm ceramic cubes of CaTiO_3_ doped with 1 wt. % Zr_2_O. At 305.5 K, the measured permittivity is 120.4 and the loss tangent is 0.007. For the first sample, we chose rubber as the intermediate layer, which has a large positive CTE of 2.42 × 10^−4^ K^−1^ and an initial width of 1 mm. Since natural materials with negative thermal expansion is rare and difficult to fabricate, we utilized heat-shrink tubing as the intermediate layer for the second sample to imitate the negative thermal expansion. The tubing has a diameter of 1.12 mm at 305.5 K, and 0.8 mm when the temperature is above 358.0 K. A third meta-atom sample with an intermediate layer of silica is also constructed. Silica has an extremely low thermal expansion of 5.3 × 10^−7^ K^−1^, which is much smaller than other materials, and the width of silica could be considered as constant. Therefore, the frequency shift of the third sample is measured as a comparative reference. The dielectric cubes were glued to the intermediate layers. Understanding that plastic or rubber components can absorb microwaves and contribute to the measured S_21_ values, and also damp the resonances, we repeated our experiments using smaller volumes of these materials on the second run. The reduced sizes in our second experiment were helpful, especially in the case of the shrink tubing. The photos of the fabricated meta-atom samples are shown in Fig. 5(e–g). This design effectively solved the problems of geometric distortion of the meta-atoms upon shrinkage of the tubing. However, there are still some discrepancies between the calculations and our measurements. These will be discussed below.

The experiment setup is composed of two WR-90 rectangular waveguides connected to the input and output of an Agilent PNA-LN5230C network analyzer, and a heating tape wound round the wave-guides and connected to an automatic temperature control heating device. During the measurements, all samples were placed in the same position in the waveguide, as shown in Fig. 4(b). As the simulation, the temperature was increased from 305.5 K to 373.0 K.

## Discussions

The S_21_ spectra of the three samples at 305.5 K and 373.0 K are shown in Fig. 5(a–c). The resonance broadening and weakening observed in the silica-containing absorbers we attribute to size nonuniformity of the dielectric cubes. Additional broadening in the other two samples’ spectra is attributable to the absorption in rubber and plastic. From the figures, we can calculate the effective TCF of the three meta-atoms, shown in Fig. 5(d). From left to right, these are 284.15 × 10^−6^ K^−1^ for the sample with intermediate layer of heat shrink tubing, 454.56 × 10^−6^ K^−1^ for silica, and 486.91 × 10^−6^ K^−1^ for rubber. Clearly the rubber produced a greater blue shift in the resonance frequency than did the silica reference. The shrink tubing yielded a diminished blue shift, though it was significantly less of a decrease than the calculations indicated. Fabrication difficulties, in particular for the sample with shrink tubing, contributed to the discrepancies from calculation. Furthermore, although the permittivity of the intermediate layers is very low compared with the dielectric cubes, its slight change with temperature could also affect the measurement results. We also consider the temperature distribution in the samples may have been nonuniform, the plastic and rubber materials being better thermal insulators than the ceramic. Blue shifts caused by the permittivity changes in the ceramics may have arisen faster than blue or red shifts owing to thermal expansion or contraction. Efforts will be made to overcome this defect by improving the coefficient of thermal conductivity of the intermediate layer.

Although our design has some deficiencies at present, this work demonstrates the feasibility of tailoring the TCF of dielectric meta-atoms by introducing a thermally expanding or contracting intermediate layer. By appropriately designing the thermal expansion of the intermediate material, dielectric meta-atoms with tunable TCF could be achieved if suitable materials and high precision manufacturing process are available. Here, we have used CaTiO_3_ ceramic cubes as the dielectric cubes, but this approach is not limited to dielectric meta-atoms. Enhanced or reduced TCF can be designed for other EM devices following a similar method.

## Conclusions

In this paper, we introduced materials with widely different CTEs into an EM meta-atom to manipulate the temperature sensitivity of its resonant frequency. Both simulation and experiment show that, by appropriately designing the meta-atom and selecting the intermediate material with suitable CTE, its microwave resonance blue shift could within limits be selected. This study covered a temperature range from 305.5 K to 373.0 K. Considering future developments, artificial materials with tunable CTE could be accessible by 3D-printing process, allowing a much broader choice for the intermediate layer material. Furthermore, this method could also be applied in the Terahertz, infrared or even optical frequencies on scaled meta-atoms.

## Methods

### Sample preparation

The ceramic dielectric was fabricated in a solid-state reaction by mixing CaTiO_3_ powders with 1 wt%ZrO_2_. The dielectric cubes were cut from a dielectric ceramic plate into dimensions of 2 mm × 2 mm × 2 mm. The meta-atoms were constructed by adhering the dielectric cubes with three different intermediate layers. The various fabricated meta-atom samples are shown in Fig. 5(e–g).

### Simulation and measurement

The meta-atom samples were measured using two WR-90 rectangular waveguides with sectional sizes of 22.86 mm × 10.16 mm × 100 mm. The other ends of the two waveguides were connected to the input and output of an Agilent Technologies N5230C vector network analyzer. The S_21_ microwave transmission spectra calculations used the CST studio suite 2016 Microwave Studio software package.

### Data Availability

The datasets generated during and analyzed during the current study are available from the corresponding author on reasonable request.
